# 3D infrared thermospectroscopic imaging

**DOI:** 10.1038/s41598-020-78887-x

**Published:** 2020-12-18

**Authors:** A. Aouali, S. Chevalier, A. Sommier, E. Abisset-Chavanne, J.-C. Batsale, C. Pradere

**Affiliations:** 1 Arts et Métiers Institute of Technology, Université de Bordeaux, CNRS, INRA, INP, I2M, HESAM, Esplanade des Arts et Métiers, 33400 Talence, France; 2 I2M TREFLE, UMR 5295 CNRS-UB-ENSAM, Esplanade des Arts et Metiers, 33405 Talence, France

**Keywords:** Imaging techniques, Infrared spectroscopy

## Abstract

This work reports a multispectral tomography technique in transmission mode (called 3DITI for 3D Infrared Thermospectroscopic Imaging) based on a middle wavelength infrared (MWIR) focal plane array. This technique relies on an MWIR camera (1.5 to 5.5 μm) used in combination with a multispectral IR monochromator (400 nm to 20 μm), and a sample mounted on a rotary stage for the measurement of its transmittance at several angular positions. Based on the projections expressed in terms of a sinogram, spatial three-dimensional (3D) cubes (proper emission and absorptivity) are reconstructed using a back-projection method based on inverse Radon transform. As a validation case, IR absorptivity tomography of a reflective metallic screw is performed within a very short time, i.e., shorter than 1 min, to monitor 72 angular positions of the sample. Then, the absorptivity and proper emission tomographies of a butane-propane-air burner flame and microfluidic perfluoroalkoxy (PFA) tubing filled with water and ethanol are obtained. These unique data evidence that 3D thermo-chemical information in complex semi-transparent media can be obtained using the proposed 3DITI method. Moreover, this measurement technique presents new problems in the acquisition, storage and processing of big data. In fact, the quantity of reconstructed data can reach several TB (a tomographic sample cube of 1.5 × 1.5 × 3 cm^3^ is composed of more than 1 million pixels per wavelength).

## Introduction

Tomography is an imaging technique that is widely used in medical imaging, geophysics, astrophysics, chemistry, material mechanics, etc. This technique allows reconstruction of the volume of an object from a series of two-dimensional (2D) measurements. Indeed, interest in tomography continues to grow, largely due to the fact that it enables the visualization of the entire volume of an object, the creation of a fine and precise representation of an object, and, finally, the study of several physical parameters. For example crack propagation, absorptivity or transmissivity fields, molecular fingerprints, or cancer diagnosis, among others^1^ can be studied using advanced tomographic techniques. The goal of this paper is to report the instrumental setup of a tomography technique and measurement procedure, especially in the middle wavelength infrared (MWIR) region. This technique can be applied in various scientific fields such as in medicine (diagnosis of cells, organs...), in engineering sciences (advanced process control for plasma torches, metrology, non-destructive control), or in chemistry (microfluidics, spectroscopy).

For years, Raman, infrared, near-infrared (NIR)^2^ and infrared spectroscopy^3^ imaging techniques have been used for the purpose of analysis of chemical compounds in solids, liquids or gases, either in reflection or transmission mode. Although NIR spectroscopy or monosensor infrared spectroscopy leads to chemical analysis in liquids or gases^4–6^, few studies exploited the potential of the imaging techniques in the MWIR region^7–9^ for solid materials. The current imaging techniques are predominantly based on spatial scanning with a monosensor, and the application domains are mainly found in biology and chemistry. On the other hand, many studies^10–13^ based on NIR diffuse tomography were performed, especially in the medical domain. Even in the NIR region, very few tomography setups used an IR camera to realize the three-dimensional (3D) fields.

In the previous works^12,13^ based on NIR diffuse tomography, it was reported that one of the most promising technique is the combination of positron emission tomography (PET) with Fourier transform infrared spectroscopy (FTIR). The high potential of PET^14^ and FTIR spectroscopy^15,16^ for separately diagnosing cancer has already been demonstrated. FTIR microspectroscopy based on a monosensor with synchrotron radiation has successfully been used to study different types of smallest tissue samples with a spatial resolution as low as 3 μm^17^. The spatial resolution achieved by PET is currently restricted to several millimetres. Although utilization of the MWIR region for the study of human tissues was not recommended^12^, mainly due to the high water absorption close to 3 μm, many other applications can find value in this IR region (non-destructive testing, study of gases, etc.). Hence, one of the advantages of the MWIR region is the possibility of monitoring temperature variations by using the proper emission. Finally, the recent progress in MWIR cameras allows us to exploit the wavelength range between 1.5 and 5.5 μm.

According to the previous literature review and to the authors’ best knowledge, few studies based on tomography has been reported in the MWIR region^18^. For this reason, it becomes important to develop new experimental tools for simultaneous tomography of chemical compounds and temperature measurements in this IR region. Such a technique would be of prime interest for complex systems where heat and mass transfer occur. Thus, the present research represents a step forward towards this goal with the development of a new tomographic benchmark in the MWIR region by using both an MWIR camera and an IR monochromator, the choice to use a monochromator instead of the FTIR is due to the fact that the signal emitted by the monochromator is much more intense than the FTIR, but less well spectrally resolved. In the first section, the complete experimental setup is described, as is the 3D reconstruction method based on Radon transform. In the second section, the method is validated on a metallic screw. It is then used on two semi-transparent media, which are a flame generated by an air-propane-butane burner (subsequently designated as a micro-torch) and on PFA tubing (filled with water and ethanol). The 3D thermal and absorptivity (defined as the amount of energy absorbed divided by the total received energy) fields from the flame and the perfluoroalkoxy (PFA) tubing are presented at the end of the paper.

This research is part of a project that aims to thermally and chemically characterize plasma torches. The main challenge is the non-contact 3D temperature field measurement for energy optimization of these torches; methods are first developed for laboratory-scale flames, and a description of the flame physics is not foreseen. Additional studies based on numerical modelling^19,20^ are being carried out by several partners in the IGAR project (see the Acknowledgement section).

## Experimental setup

**Figure 1:**
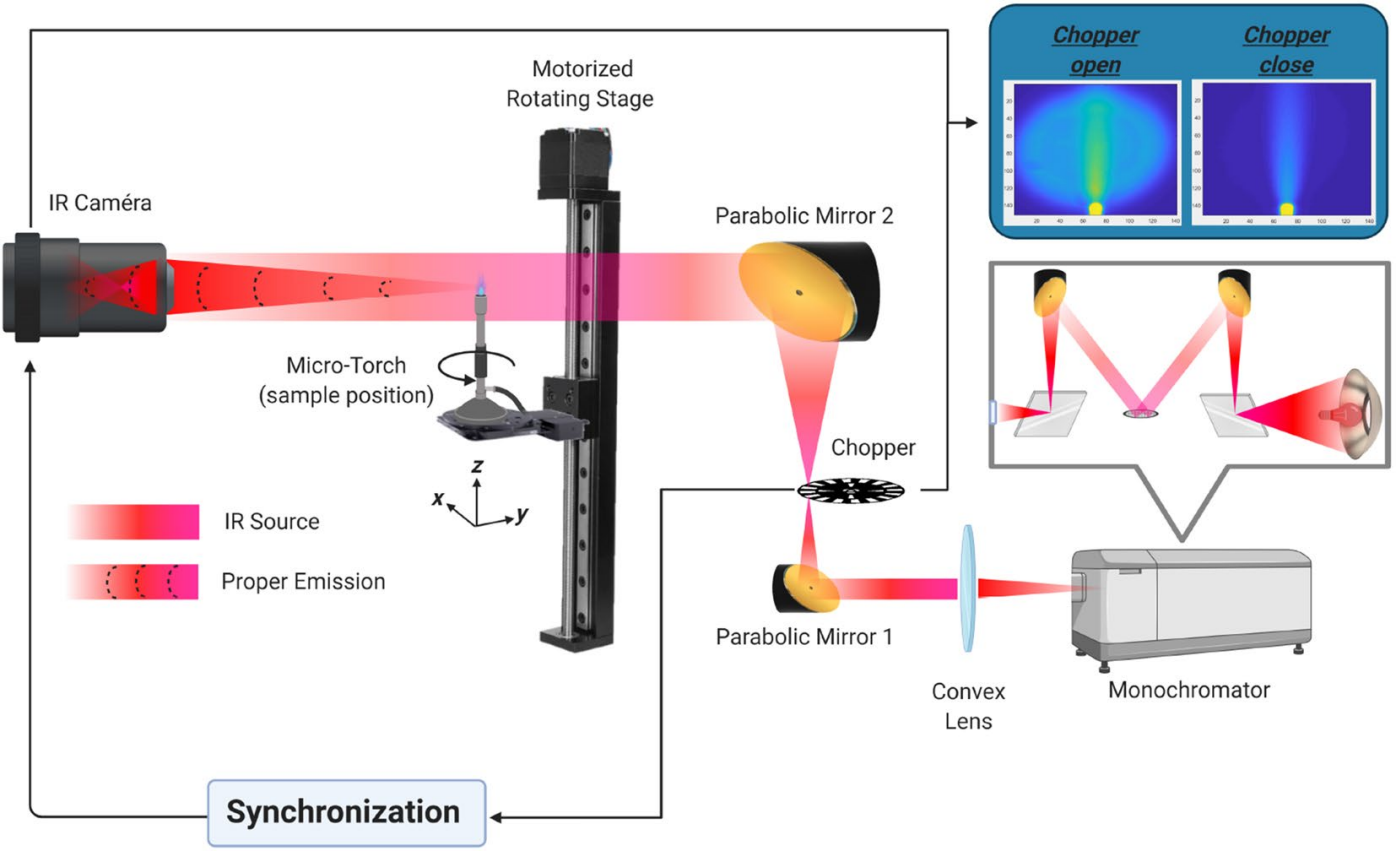
Schematic of the experimental setup. This illustration is made for a micro-torch. (Created with https://BioRender.com).

The system, presented in Fig. 1, is composed of an IR camera (FLIR SC7000) and a monochromator used to select the excitation wavelength (Bentham, TMC 300). The source of the monochromator is a black body lamp (Nernst and halogen) emitting from 400 nm to 20 μm with three gratings to tune individual wavelengths. The gratings are mounted on a rotated turret controlled by mechanical motors to sweep all of the wavelengths. Order sorting filters are used to transmit only the first diffraction order. For this study, only one blazed grating with 300 grooves was used to scan over a broad IR band ranging from 1.5 to 5.5 μm at a 10 nm step. Two parabolic mirrors and one germanium plano-convex lens control the optical path of the beam inside the spectrometer. Moreover, a chopper can be used to periodically modulate the monochromatic emitted light. The signal at the position of the slit (square of 1 cm^2^) is then collimated with the germanium plano-convex lens and expanded by a magnitude of 3 by the two parabolic mirrors with respective focal lengths of 50.8 mm and 152.4 mm^21^ to obtain a homogeneous incident source at the location of the sample. The transmitted light passing through the sample is recorded by the IR camera used as a sensor. The IR camera has an InSb focal plane array (FPA) composed of 76,800 individual sensors (FPA composed of 240 × 320 pixels and pitch size of 25 μm × 25 μm) with a 50 mm objective and an MWIR F/2 focal length (spatial resolution of 167 μm per pixel). The wavelength range of the camera includes 1.5 μm to 5.5 μm. The frame rate acquisition was set to 100 Hz. The photon count of the emitted intensity was detected with a 14-bit ADC (Analog to Digital Converter). The infrared spectra were acquired using the monochromator synchronised with the IR camera with a homemade LabVIEW and Matlab software to control the acquisition frequency and scan the wavelength over the domain of interest. The same software also facilitates the frame recording and data saving in appropriate formats for post-processing.

**Figure 2:**
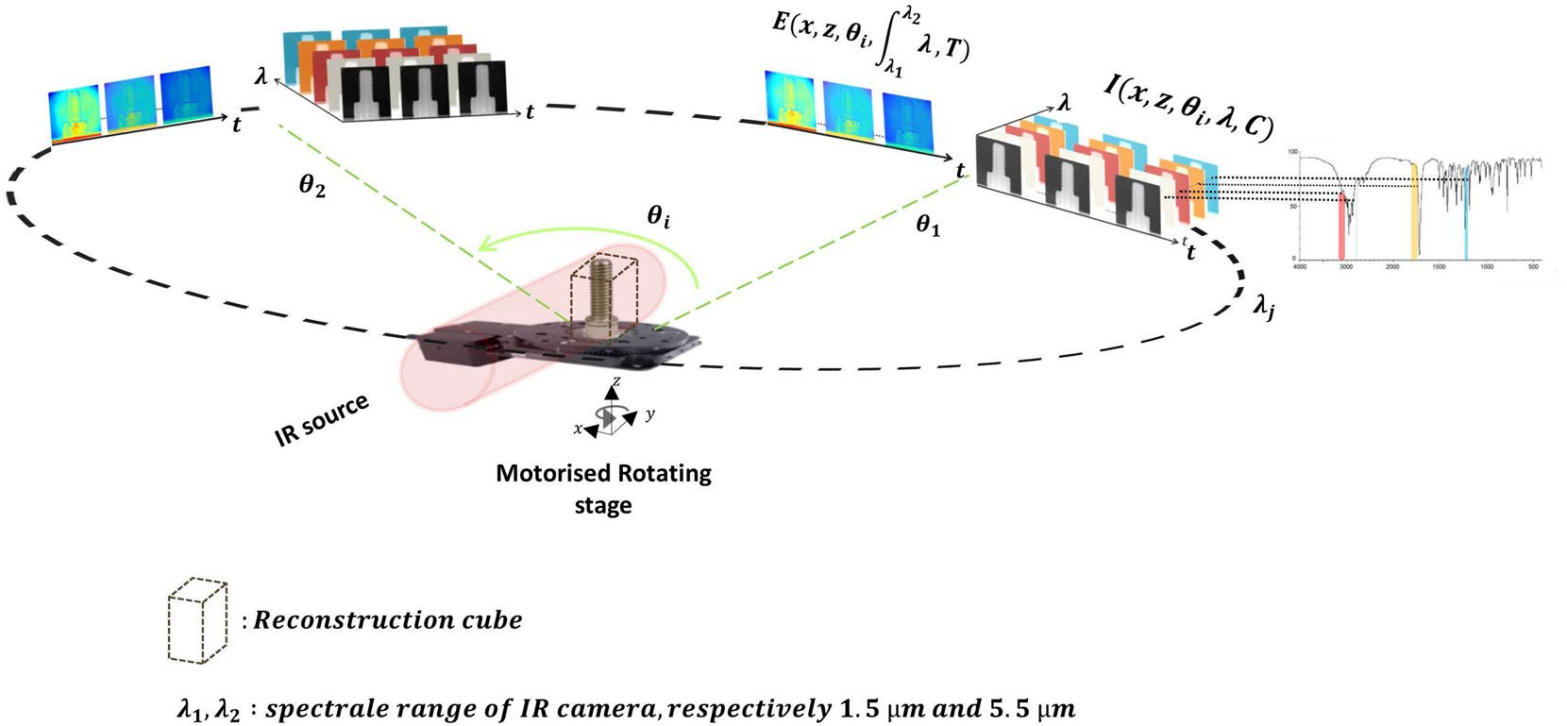
Scheme of the 3DITI measurement principle. (Created with Powerpoint 2013, www.microsoft.com).

The sample (for example, the micro-torch in Fig. 1) is mounted on a rotary stage (X-RST Zaber series) to experimentally acquire the Radon transform projected images of the sample. As shown in Fig. 2, for each angular position, both the proper emission and spectral signal are recorded (see Eq.1). This can be performed for all IR wavelengths in the range between 1.5 and 5.5 μm. In this paper, only one wavelength of 4 μm was used for the screw and the flame because it is the peak intensity delivered by the monochromator and a good wavelength with respect to flame gas transparency^22^. In the second step, the multi-spectral aspect of the setup is demonstrated using the semi-transparent PFA tubing imaged at two wavelengths.

The rotary stage can reach speeds of up to 24°/s and torques up to 10 Nm, which will ensure rapid acquisition. To avoid vibrations and sample movements, a rotation speed corresponding to 4 rpm was applied. This makes it possible to achieve the 72 angular positions in approximately 1 min. Here, on the basis of the fastest spectroscopy systems proposed by Süss et al.^23^, the system offered by Süss et al. allows 76,923 scan/s with a monosensor, which is on average already 1000 times faster than conventional systems. Thus, a classic small-format IR camera can acquire 1 image of 320 × 256, i.e., 81,920 pixels, in 100 μs, as presented in^8^. The wavelength scanning of the monochromator in the 1.5 to 5.5 μm range is performed at a speed of 0.5 wavelength/s, or within approximately 3 min, to create the full spectrum. Consequently, conduction of tomography for 72 angular positions will be done quickly in less than 1 min, which makes it possible to carry out a dynamic tomographic study. Moreover, the recent work of^21^ showed that the combination of a commercial FTIR type system and an IR camera allows us to conduct a scan of 1000 wavelengths for an image size of 80,000 pixels in only a few seconds.

Following the methodology described in previous papers^8,24^, the acquired signal $S$ from the contributions of two distinct signals is given by:

1 $$\begin{aligned} S(x,z,\lambda ,C,T)=I(x,z,\lambda ,C)+E(x,z,\lambda ,T), \end{aligned}$$

where $I$ and $K$ are the transmitted IR signal and the proper emission, respectively, $x$ (mm) and $z$ (mm) are spatial coordinates, $C$ (mol m^−3^) is the molar concentration, $\lambda$ (μm) is the wavelength, and $T$ (*K*) is the temperature. Both signals are hypercubes of thermal and spectral images, as illustrated at the top of the Fig. 2. An interesting feature of the experimental setup is that both contributions can be measured independently and quasi-simultaneously by closing or opening the chopper with a synchronized internal trigger of the IR camera.

## 3DITI procedure

### Acquisition principle

**Figure 3:**
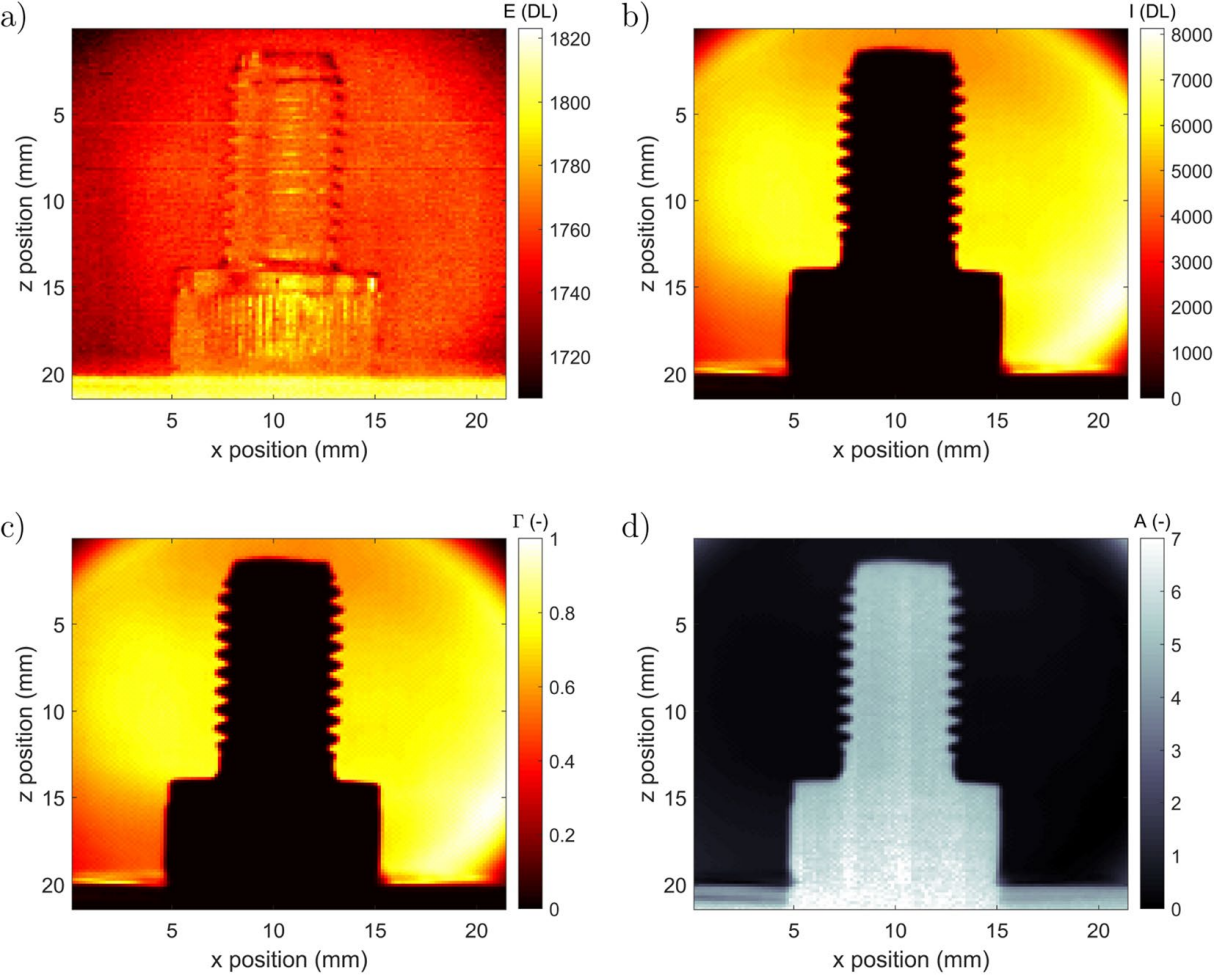
Measured images at integration time IT = 200 μs and $\theta$ = 46°: (**a**) proper emission $E(x,z,\lambda ,T)$, (**b**) spectral signal $I(x,z,\lambda ,C)$ at $\lambda$ = 4 μm, (**c**) calculated transmittance $\Gamma (x,z,\lambda ,C)$ from Eq. 3 and (**d**) calculated absorbance $A(x,z,\lambda ,C)$ from Eq. 4. (Created with MATLAB 2020a, https://mathworks.com).

To validate the tomography technique and measurement procedure, an ideal and reflective sample (metallic screw, M6) of 12.5 mm height with a diameter of 6 mm was chosen. As depicted in Fig. 1, the sample is mounted on a rotary stage to experimentally acquire the Radon transform projected images of the sample. As shown in Fig. 2, for each angular position, both the proper emission (Fig. 3a) and spectral signal (Fig. 3b) are recorded (see Eq.1). This can be performed for the entire IR wavelengths in the range between 1.5 and 5.5 μm.

The transmitted intensity signal *I* is expressed as follows:

2 $$\begin{aligned} I(x,z,\theta _i,\lambda ,C)=\Gamma (x,z,\theta _i,\lambda ,C)I_{0}(x,z,\lambda ) , \end{aligned}$$

where $I(x,z,\theta _i,\lambda ,C)$ corresponds to the transmitted signal images as a function of the wavelength and the concentration of the sample or its chemical composition. $\Gamma (x,z,\theta _i,\lambda ,C)$ is the transmittance of the sample, and $I_{0}(x,z,\lambda )$ is the intensity of the incident signal from the monochromator. Therefore, assuming the Beer–Lambert law for the source absorption, the transmittance is defined as:

3 $$\begin{aligned} \Gamma (x,z,\theta _i,\lambda ,C)= \exp \left[ -\int _0^{Ly}\mu (x,z,y,\lambda ,C)dy\right] =\dfrac{I(x,z,\theta _i,\lambda ,C)}{I_{0}(x,z,\lambda )}, \end{aligned}$$

where $\mu (x,z,y,\lambda ,C)$ (m^−1^ mol^−1^) is the absorptivity (molar attenuation coefficient) as a function of the chemical composition of the sample and $L_y$ (m) corresponds to the thickness of the sample in the *y*-direction. In this case, a metallic sample will appear as a completely opaque medium in terms of attenuation even if, from a physical point of view, it acts as a perfect mirror with strong reflective properties. Therefore, from Eq.3, the absorbance is expressed as follows:

4 $$\begin{aligned} A(x,z,\theta _i,\lambda ,C)=-log[\Gamma (x,z,\theta _i,\lambda ,C)]=\int _0^{Ly}\mu (x,z,y,\lambda ,C)dy. \end{aligned}$$

Based on the images presented in Fig. 2 and the relation established in Eq.4, the absorbance as a function of the angular position can be calculated from the measurement of the intensity signal (from Eq.1) and is presented in Fig. 3d. The incident intensity signal $I_{0}(x,z,\lambda )$ was measured separately.

The reconstruction of the 3D absorptivity field is performed by the back-projection method based on the Fourier transform^25^; for this purpose, the following back-projection operator is used:

5 $$\begin{aligned} {\mathcal {B}}[\mu (x,z,y,\lambda ,C)]= \int _0^\pi \tilde{A}(x\cos \theta + z\sin \theta ,\theta ,\lambda ,C) d\theta , \end{aligned}$$

where $\tilde{A}$ designates the Fourier transform in polar coordinate of absorbance *A*. Finally, the 3D absorptivity field is reconstructed using Eq.6:

6 $$\begin{aligned} \mu (x,z,y,\lambda ,C)= {\mathcal {B}}\left\{ {\mathcal {F}}^{-1}\left[ {\mathcal {F}}[\tilde{A}(x,z,\theta ,\lambda ,C)].|{\mathcal {W}}|\right] \right\} , \end{aligned}$$

where ${\mathcal {F}}$ designates the Fourier transform, and $|{\mathcal {W}}|$ is a ramp filter^25^.

### Inverse Radon reconstruction

The first images obtained using the metallic screw are presented in Fig. 3. One can observe in Fig. 3.a that due to the short integration time used for the measurements and the high reflectivity (low emissivity) of metal, the thermal field quality is not ideal. Nevertheless, one can note the ability of the setup to simultaneously measure proper emission (at the surface of the sample) and the spectral transmitted signal. In Fig. 3b, the Radon projection of the spectral signal exhibits a very good quality and an important signal (an approximately 8000 digital level). From these images, the transmittance fields can be calculated from Eq.3 (Fig. 3c), as can the absorbance (Fig. 3d) from Eq.4. It is clearly observed on the transmittance image (Fig. 3c) that the metallic sample used in the study acts as a perfect reflective media with transmittance close to zero. In contrast, the absorbance image in Fig. 3d shows a value close to zero for the incident beam alone and a value close to 7 (maximum measurable with the device) at the screw position.

**Figure 4:**
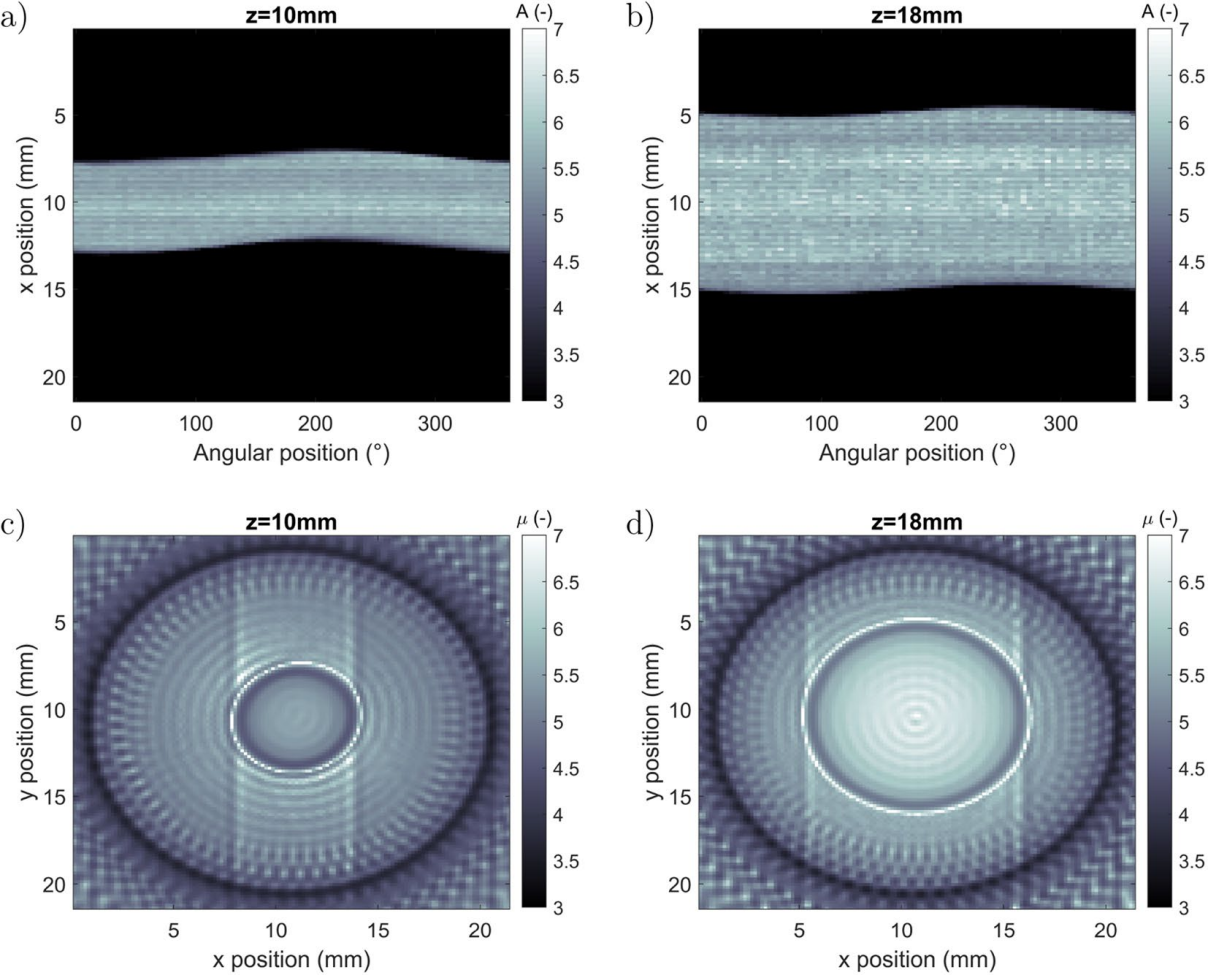
(**a**) Sinogram of the absorbance extracted at z = 10 mm (thread of the screw) over all of the angular positions, (**b**) sinogram of the absorbance extracted at z = 18 mm (head of the screw) over all of the angular positions, (**c**) reconstructed slice (x and y) at z = 10 mm for the thread of the screw and (**d**), reconstructed slice (x and y) at z = 18 mm for the head of the screw. (Created with MATLAB 2020a, https://mathworks.com).

From the measurements presented in Fig. 3, back-projection reconstruction^25–27^ using inverse Radon transform was implemented. For this, the sinograms corresponding to one line (at one *z*-location) of the image in Fig. 3d as a function of angular position are presented in Fig. 4a and b for $z=10$ mm and $z=18$ mm, respectively. In these figures, the sizes of the two z-planes corresponding to the thread of the screw (Fig. 4a) and the head of the screw (Fig. 4b) indicate the different sizes.

From this sinogram representation of all z-positions, the different slices perpendicular (along the x and y directions) to the measurement direction can be calculated by the inverse Radon transform^26,27^ using Eq. 6. Examples of the reconstructed slices are shown in Fig. 4c and d with the true dimensions of the screw.

### Validation of the method

**Figure 5:**
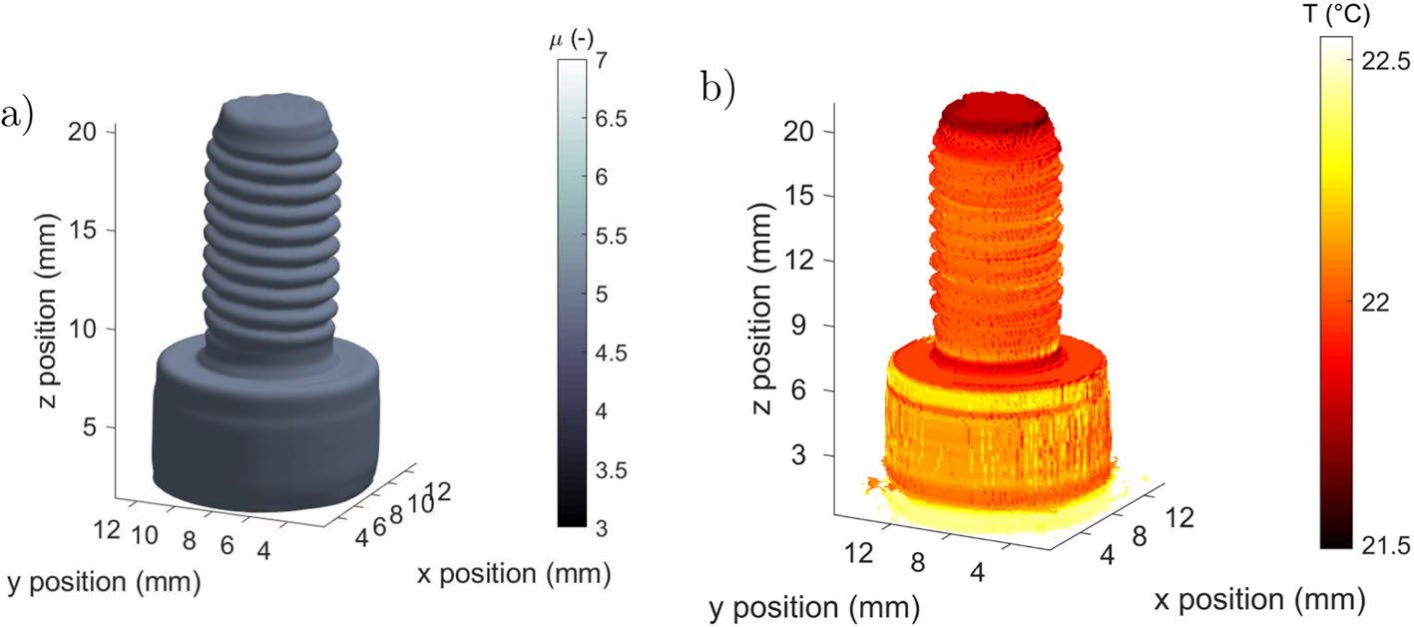
Reconstructed three-dimensional maps of the M6 screw sample: (**a**) absorptivity tomography at $\lambda$ = 4 μm and (**b**) temperature map in °C at the surface of the sample. (Created with MATLAB 2020a, https://mathworks.com).

The complete computational tomographies of the sample were acquired with inverse Radon processing over all of the *z*-direction. These results are presented in Fig. 5a for the absorptivity and in Fig. 5b for the temperature. These tomographies highlight the perfect shape and appearance of the reconstructed screw. One can observe the good spatial resolution (8.25 × 10^−3^ mm^3^ per voxel) of the tomography, especially in the reconstruction of the thread. The results validate the proposed methodology to generate 3D absorptivity volumes.

With the ability of the device to perform simultaneous spectral and thermal measurements, the proper emission fields measured at the surface of the screw can also be recalculated as shown in figure 5.b. With a calibration performed at room temperature with a black body reference, the proper emission *E* was converted to temperature field, this calibration is only valid in the case of the screw, because it is static and the emissivity is homogeneous and constant. As expected, the 3D temperature field of the screw shows a homogeneous temperature close to room temperature, i.e., 22 °C.

## Results and discussion in semi-transparent media

### Microtorch flame

**Figure 6:**
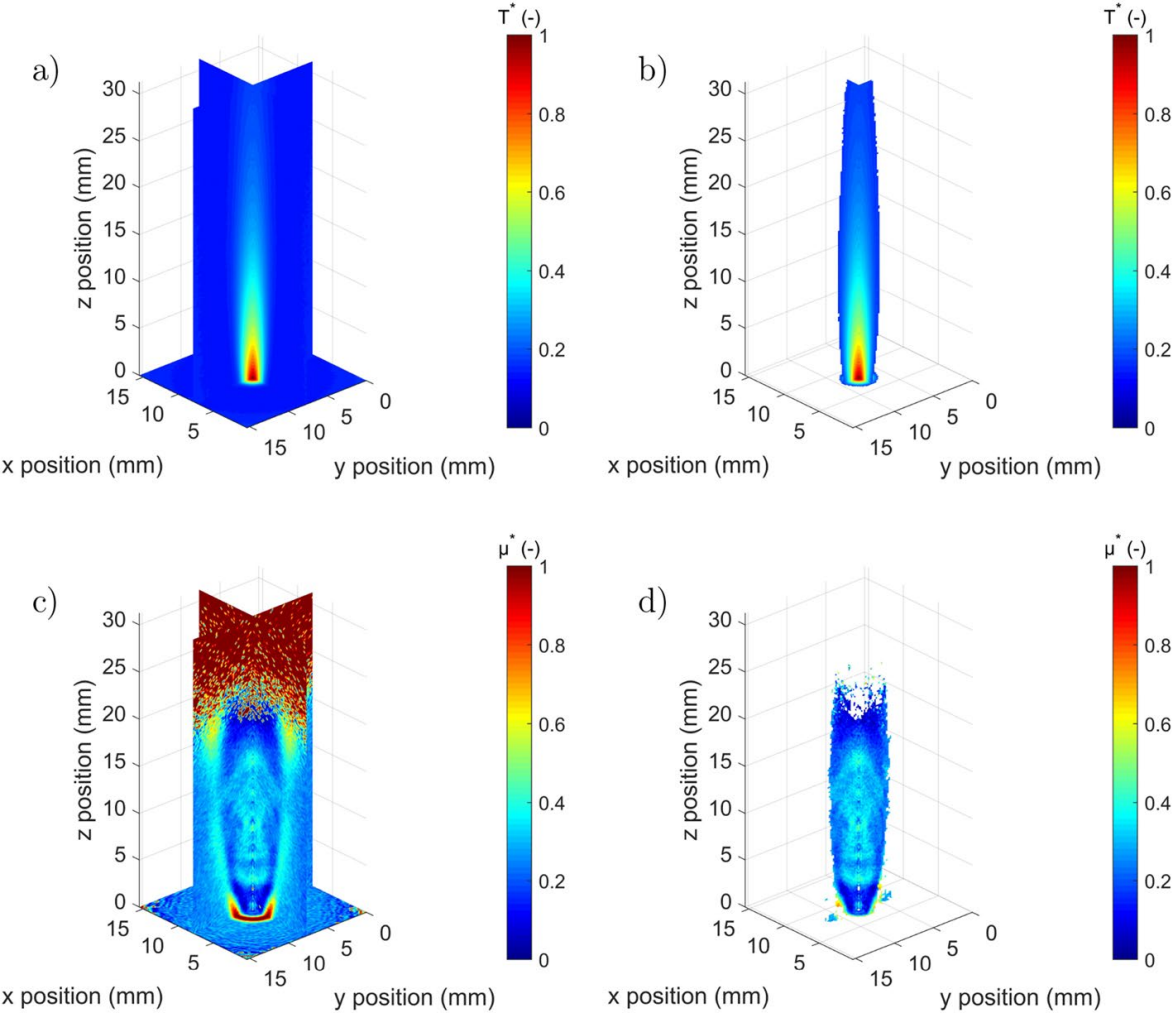
Dimensionless reconstructed three-dimensional maps of the flame: (**a**) 3D proper emission map, (**b**) 3D proper emission map (background removed) and (**c**) 3D absorptivity map at $\lambda$ = 4 μm, (**d**) 3D absorptivity map at $\lambda$= 4 μm (background removed). (Created with MATLAB 2020a, https://mathworks.com).

The second experiment carried out is 3DITI of a flame generated by a micro-torch, where both the 3D absorptivity and proper emission fields are measured. The difficulty of such measurements lies in the extreme conditions of temperature and convection. Thus, for a complete quantitative measurement^24^, it is necessary to locally measure the 3D emissivity fields via a spectral approach and to set up iterative methods for estimating the absolute temperature from the emissivity measurement coupled with a transmission calibration procedure. A specific procedure will be developed at a later stage for this type of measurement, but it is currently outside the scope of the present paper, which aims to highlight the ability to perform 3D tomographic imaging of an IR semi-transparent medium. For this purpose, the fields obtained in Fig. 6 are dimensionless; i.e., they were normalized by the maximum of each of the obtained raw fields.

To realize the complete measurements, the following steps are performed: (i) the micro-torch is positioned with the rotation plates at a given angular position, as shown in Fig. 2; (ii) taking into account the time-varying character of the flame, a blue, oxygen-rich flame is generated by the micro-torch, and a one-second film at 200 fps is produced (synchronization with the chopper and averaging the different images of the film to extract the images of the proper emission and the spectral signal); (iii) the different angular positions, 72 in total, are chained together; and (iv) the micro-torch is turned off in order to acquire the incident intensity signal $I_{0}(x,z,\lambda )$ measured at $\lambda$ = 4 μm. This complete 3D scan is performed in approximately 3 min, which is three times longer than the scan for the previous example with the screw. This is due to the fact that a film is produced for every angular position to average the images and reducing the noise. In fact, a rotation speed corresponding to 3 rpm has been applied using the step-scan mode. This makes it possible to achieve the 72 angular positions in approximately 1 min, plus the acquisition time at each position. Refer to section “Experimental setup” for the detailed description of the system acquisition speed. From these data, the inverse Radon processing described in section “3DITI procedure” is applied in order to reconstruct the 3D maps of Fig. 6.

The results of the 3D flame reconstruction are presented in Fig. 6. The shape of the two fields shows that the absorptivity is wider than the proper emission. It can also be noted that the absorptivity field is limited at the top by a noisy red background corresponding to the region outside of the IR beam. Thus, from the point of view of 3D mapping, it is clear that the quantities of Eq. 1 relating to the proper emission Fig. 6a and b and the absorptivity Fig. 6c and d do not seem to be sensitive to the same parameters. The difference essentially lies in the shape and homogeneity of the two maps. The proper emission image, rather proportional to the flame temperature, is very intense at the heart of the flame and stretches into a jet shape. Conversely, the field proportional to the absorptivity is much more spread out and homogeneous. At this stage of the analysis, a description of the physics of the flame is not foreseen. However, these data provide experimental validation for future models of laminar flames.

The comparison of the homogeneity of the two fields shows that the spatial distribution of the absorptivity does not necessarily follow that of the proper emission. One explanation for this physical phenomenon lies in the physical and chemical nature of the flame, which influences the radiative properties of the molecules, in particular the absorptivity.

As mentioned before, the estimation of the true flame temperature necessitates the measurement of the emissivity field. Using a method similar to the one described in this paper and the property that semi-transparent bodies (a flame in our case) do not reflect IR radiation^28^, one can simultaneously access the transmissivity and absorptivity of the body through the thermospectroscopic method. This is a direct possible extension of the method proposed here for true 3D flame temperature measurement.

### Microfluidic PFA tubing

**Figure 7:**
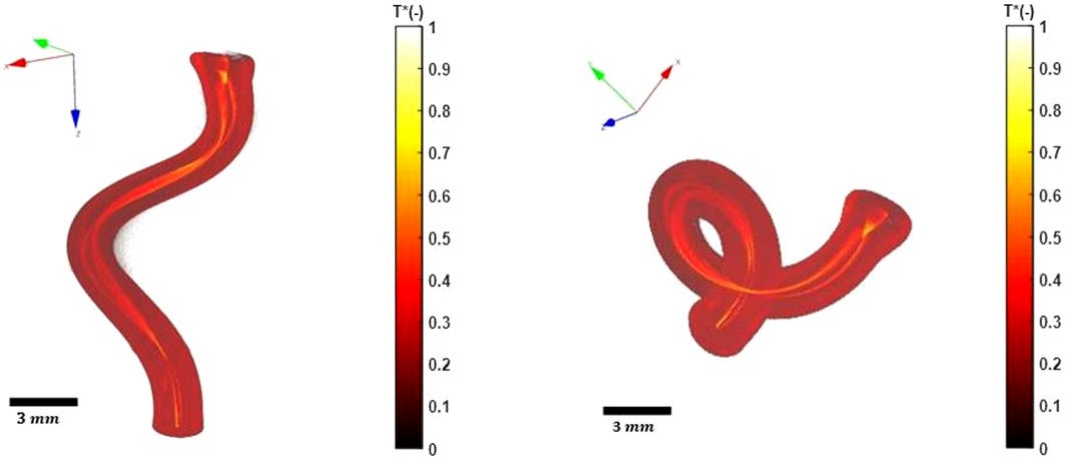
Dimensionless reconstructed three-dimensional proper emission maps of the PFA tubing filled with water and ethanol. (Created with MATLAB 2020a, https://mathworks.com).

**Figure 8:**
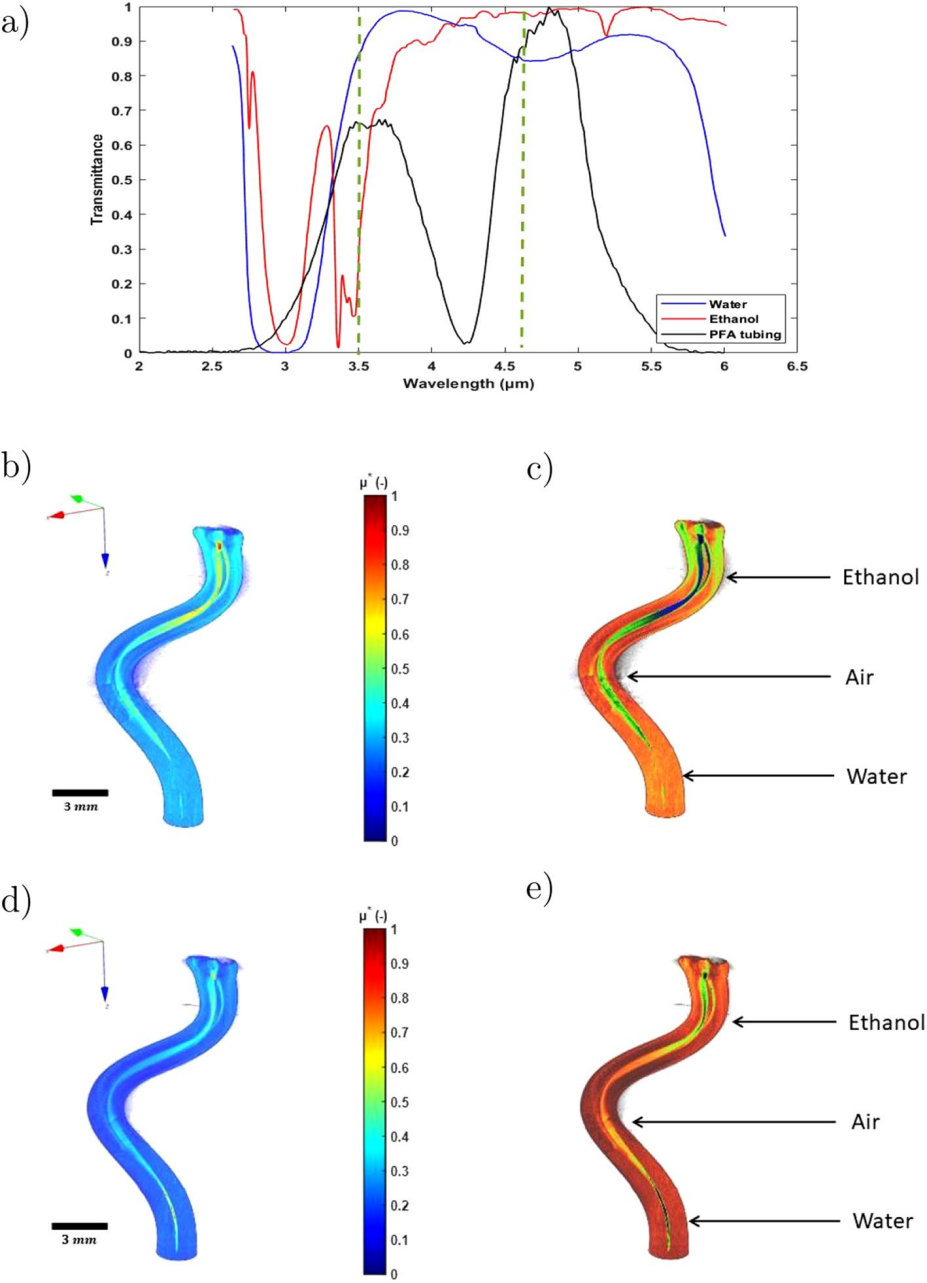
(**a**) Transmittance of water, ethanol and PFA tubing. Dimensionless reconstructed three-dimensional absorptivities maps of the PFA tubing: (**b**) 3D absorptivity map at $\lambda$= 3.5 μm, (**c**) 3D absorptivity map at $\lambda$= 3.5 μm with colored segmentation for each media, (**d**) 3D absorptivity map at $\lambda$= 4.6 μm, (**e**) 3D absorptivity map at $\lambda$= 4.6 μm with colored segmentation for each media. (Created with MATLAB 2020a, https://mathworks.com).

The aim of the third experiment carried out is the 3DITI of a semi-transparent and spectrally heterogeneous medium which has a complex and non-axisymmetric shape. The sample chosen is a spiral-shaped PFA tubing ($\phi _{ext}=1.8$ mm, $\phi _{int}= 0.8$ mm). Taking into account the transmittance of the PFA tubing (see Fig. 8a), two liquids (water and ethanol) with interesting transmittance spectra were chosen (see Fig. 8a). Thus, the PFA tubing is respectively filled (from top to bottom) with ethanol, an air bubble and water to form an heterogeneous medium that reacts differently depending on the wavelength. The 3D fields of the proper emission and the absorptivity at these wavelengths was measured and presented in Figs. 7 and 8, respectively.

Figure 7 shows the dimensionless 3D reconstruction of the proper emission (radiative flux proportional to the product of the temperature and the emissivity of the sample) of the PFA tubing. The spiral (non-axisymmetric) shape of the PFA tubing is perfectly reconstructed, which demonstrates that the proposed method is not limited only to axisymmetric samples. In addition, a proper emission contrast is visible in Fig. 7. This observation can only be explained by a variation of the emissivity between the different components (PFA tubing, water, air and ethanol) as all the material can be considered isothermal at the room temperature.

Figure 8a shows the IR transmittance spectra of the different components in the spectral range 2–6 μm. The analysis of the transmittance spectra of the different media allows the identification of two wavelengths of interest which ensure a contrast in terms of absorptivity, i.e. 3.5 μm and 4.6 μm.

A comparison of the 3D absorptivity maps at two different wavelengths (3.5 μm and 4.6m) is illustrated in Fig. 8b and d, respectively. These results show that the 3D map at 3.5 μm highlights a contrast between the absorptivity of the different components, i.e., ethanol absorbs IR radiation more than the others, and that water is almost transparent. In contrast, the 3D map at 4.6 μm shows that all the components absorb IR radiation at slightly different rates. These observations are concordant with the analysis of the transmittances of the different components which are represented in Fig. 8a.

Figure 8c and e shows respectively the 3D absorptivity maps of the two wavelengths with colored segmentation. Color segmentation in the images is obtained by modifying the RGB color map of the figure. This modification consists in creating a Gaussian function centered around the maximum absorptivity value of each medium, and the standard deviation is chosen as a function of the desired color range linked to the medium absorptivity range. These results complement and reinforce the previous observations by highlighting more clearly the absorptivity contrasts of the whole heterogeneous media.

The work carried out at a single wavelength for the flame and the screw and at two wavelengths for the PFA tubing, highlights the system’s ability to perform diffuse tomography on a semi-transparent medium in a rapid recording time of less than 1 min for the screw and the PFA tubing (for each wavelength), and less than 3 min for the flame. The time to perform the experimental Radon transform is approximately 1 min (for one wavelength) with more than 1 million pixels in the 3D reconstructed cube. Consequently, there is no obstacle to realizing 5D tomography of chemical compounds (hypercube of five dimensions: three for space, one for time and one for wavelength) of an MWIR semi-transparent system.

**Figure 9:**
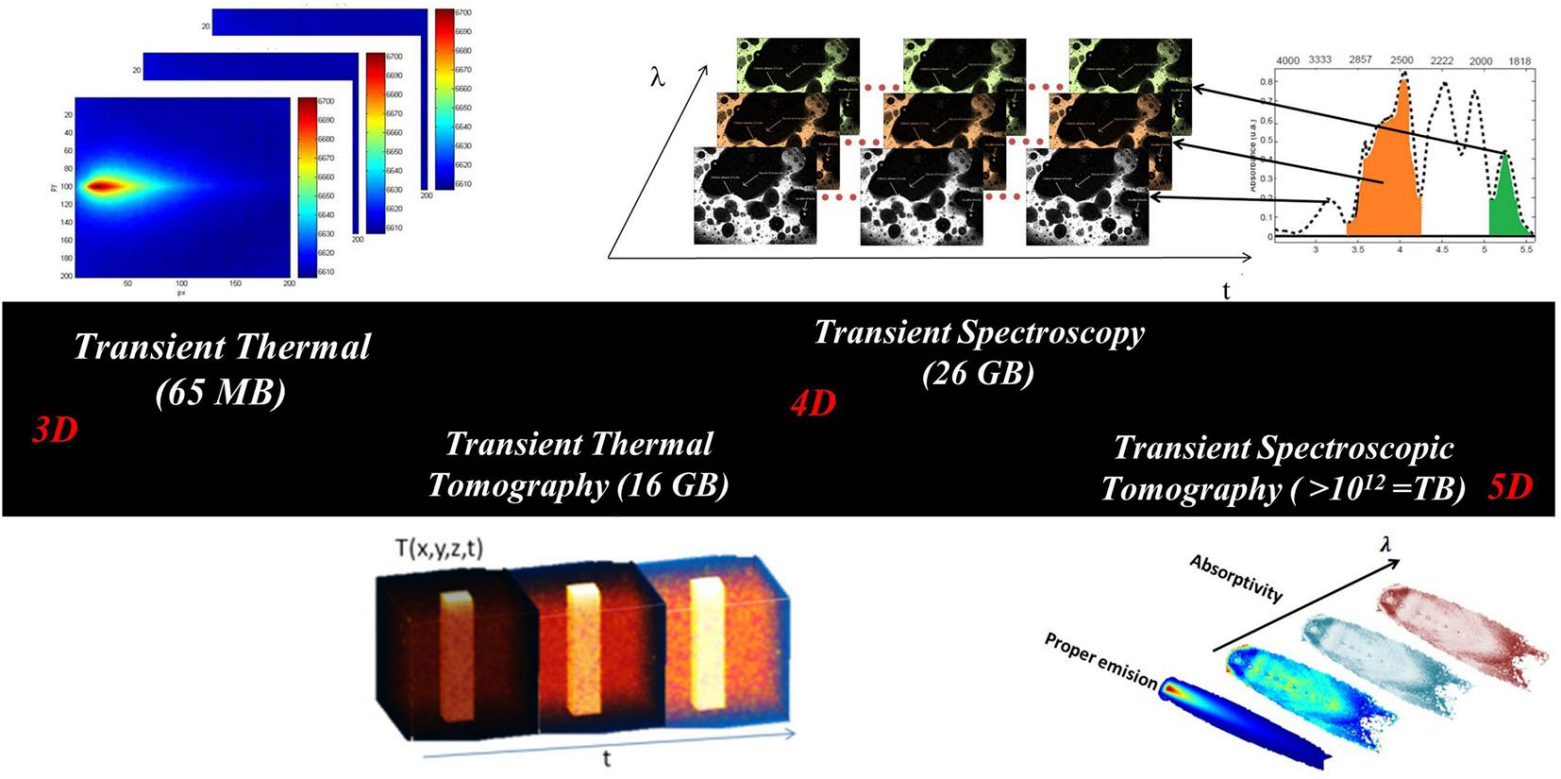
Data generated with IR spectroscopic imaging for different transient diffusion problems. (Created with Powerpoint 2013, www.microsoft.com).

In this study, the complete multispectral tomography of an object cannot be acquired with the equipment at our disposal. Indeed, a spatial cube of more than 5.8 million pixels, created at 200 fps and with 100 wavelengths in the MWIR range, would represent more than 1.65 TB ($200 \times 320 \times 256 \times 72 \times 100 \times 14 = 1.65$ TB) of data to be stored every 3 min (Fig. 9).

## Conclusion

To conclude this work, it was shown that the 3DITI technique is a powerful tool to measure 3D fields of semi-transparent IR media. In this paper, the methodology and the design of the system were described. The most important point is that the introduction of the FPA sensor into IR spectroscopy allows for ultra-fast measurements of 2D spectral fields. Then, inverse Radon transform based on these 2D measurements enabled 3D field reconstruction. The results were validated using a metallic sample, presented here as the reference. The development of the measurement and back-projection method was used to highlight the spatial quality of the images. Finally, a second tomography study was carried out on heterogeneous media such as a micro-torch flame and PFA tubing to image, for the first time, the 3D absorptivity and proper emission fields of such heterogeneous and semi-transparent to IR materials. This research provides new perspectives in the domain of IR imaging spectroscopy, as well as in the domain of hypercube and big data processing with many expected application in the fields of microfluidic, biology and living tissue.
